# Effect of Kencur (*Kaempferia galanga L.*) Ethanolic Extract Treatment on Cerebral Caspase-3 Expression in Traumatic Brain Injury Rat Models

**DOI:** 10.21315/mjms2024.31.2.5

**Published:** 2024-04-23

**Authors:** Army Pambudi Suryo, Rizki Meizikri, Tedy Apriawan, Agus Turchan, Lucia Yovita Hendrati, Abdul Hafid Bajamal, Muhammad Arifin Parenrengi, Budi Utomo, Dyah Fauziah, Priangga Adi Wiratama

**Affiliations:** 1 Department of Neurosurgery, Faculty of Medicine, Universitas Airlangga - Dr. Soetomo Academic General Hospital, Surabaya, Indonesia; 2 Department of Neurosurgery, Universitas Airlangga Academic Hospital, Surabaya, Indonesia; 3 Department of Epidemiology, Population, Biostatistics and Health Promotion, Faculty of Public Health, Universitas Airlangga, Surabaya, Indonesia; 4 Department of Public Health Science and Preventive Medicine, Faculty of Medicine, Universitas Airlangga, Surabaya, Indonesia; 5 Department of Clinical Pathology, Faculty of Medicine, Universitas Airlangga - Dr. Soetomo Academic General Hospital, Surabaya, Indonesia

**Keywords:** traumatic brain injury, Kaempferia galanga L., apoptosis, caspase-3, traditional medicine, Kencur

## Abstract

**Background:**

Traumatic brain injury is one of the most common forms of trauma and causes significant morbidity and mortality. Kencur (*Kaempferia galanga* L.) ethanolic extract is known to contain substances that could theoretically inhibit unfavourable cellular processes, including oxidative stress and inflammation. This research aimed to study Kencur’s anti-apoptosis activity through the inhibition of caspase-3.

**Methods:**

This is a true experimental post-test-only group design study, using male Wistar rats (*Ratus novergicus*) with weight-drop-induced traumatic brain injury. The subjects in this study were divided into four groups: two Control groups (Groups A and B) and two Therapy groups (Groups C and D). Groups C and D differed in the dose of Kencur ethanolic extract administered (600 mg/kgBW/day and 1,200 mg/kgBW/day, respectively). The Therapy groups were then subdivided into those receiving therapy for 24 h (C-24 and D-24) and those receiving therapy for 48 h (C-48 and D-48). Caspase-3 expression in brain tissue was evaluated at the end of the therapy using immunohistochemistry. All groups were subjected to a Kruskal-Wallis comparison test and the investigation continued with a Mann-Whitney U test to compare the two groups.

**Results:**

In traumatic brain injury rat models treated with *Kaempferia galanga* L. ethanolic extract at doses of 1,200 mg/kgBW/day within 48 h of therapy (D-48) compared to those who were not treated, there was a significant change in the cerebral expression of caspase-3 (*P* = 0.016). There was also a significant difference between the two doses of intervention (C-24 at 600 mg/kgBW/day and D-48 at 1,200 mg/kgBW/day; *P* = 0.016).

**Conclusion:**

With a minimum of 48 h of treatment split into two doses, Kencur (*Kaempferia galanga* L.) ethanolic extract can decrease caspase-3 expression in rats with traumatic brain injury.

## Introduction

Traumatic brain injury is among the main causes of death and disability in trauma cases. The majority of traumatic brain injury survivors live with disabilities and with diminished productivity (1). This issue greatly affects not only the patient’s health but also the well-being of their families (2). Cysteine-aspartic acid protease-3 (caspase-3) is a coding protein that plays a role in the process of executing cell apoptosis. Increases in brain metabolism and cerebral blood flow occur concomitantly with increases in inflammatory response and activation of free radicals in traumatic brain injury, which then triggers the caspase pathway and ultimately induces apoptosis (3).

Neuroprotection against apoptosis can be accomplished by inhibiting apoptotic pathways and/or stimulating survival pathways. Kencur (*Kaempferia galanga* L.) is a stemless, rhizomatous, aromatic, perennial and indigenous herb. It is native to India and Southeast Asian countries such as Indonesia and Malaysia (4). Kencur contains active ingredients that have anti-inflammatory properties (ethyl p-methoxycinnamate), antioxidants from the class of flavonoids (tetrahydroxyflavones), ascorbic acid and saponins (5–7). The active ingredients of *Kaempferia galanga* L. inhibit free radicals and inflammatory responses that trigger apoptosis through the caspase pathway and ultimately inhibit apoptosis. This study aimed to investigate the prospect of Kencur in inhibiting apoptosis by observing its effect on caspase-3 expression in rat models of traumatic brain injury.

## Methods

### Animal Model

The in vivo model of traumatic brain injury in this study was male Wistar rats (*Ratus novergicus*) aged 2.5 months old–3 months old and weighing 300 g. Samples were allocated to each group by the veterinary’s assistant, who was blind to the grouping scheme of this study and then put randomly in unlabeled cages.

The number of samples for the hypothesis test formula:


$$n=\frac{2\sigma^{2}{\left[Z_{1-\alpha /2}+Z_{1-\beta }\right]}^{2}}{{\left(\mu_{1}-\mu_{2}\right)}^{2}}$$

*n* = number of replications
*Z*_1–_*_α_*_/2_ = standard value of normal distribution at α 0.05 (1.96)
*Z*_1–β_ = standard value of normal distribution at β 0.01 (1.28)
σ = standard deviation (1.08)
μ_1_ = mean group with treatment (3.40)
μ_2_ = mean control group (0.42)

Sample size:


$$\begin{array}{l} n=\frac{2\times {\left(1.08\right)}^{2}{\left(1.96+1.28\right)}^{2}}{(3.40-0.42)2}=\frac{25.58}{8.88}\\ =2.8\sim 3 \end{array}$$

The number of replications calculated by the formula for each group was three samples.

Number of replications with correction factors:


$$\begin{array}{l} n^{\prime }=n\times 1/(1-f)\\ =3\times 1/(1-0.3)\\ =4.3\sim 5 \end{array}$$

*n*’ = number of replications after correction
*f* = correction factor according to risk of treatment (0.3)

**Figure 1:**
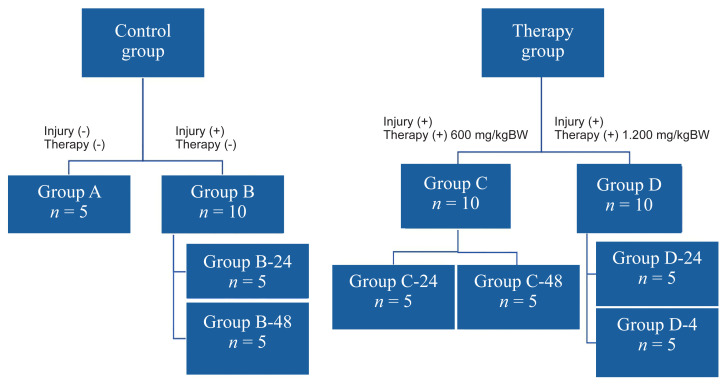
Recruitment and selection

The number of Wistar rats (*Ratus novergicus*) required for each group after the correction factor was 5. The subjects were divided into two major groups: i) the Control groups (Group A, without traumatic brain injury and not receiving Kencur ethanolic extract; and Group B, with traumatic brain injury but not receiving Kencur ethanolic extract), and ii) the Treatment groups (Groups C and D: those with traumatic brain injury and given Kencur ethanolic extract). Groups C and D differed in the dose of Kencur ethanolic extract administered (600 mg/kgBW/day and 1,200 mg/kgBW/day, respectively). Group B was divided into two subgroups, terminated at 24 h and at 48 h after the onset of trauma (addressed as B-24 and B-48 from here onwards). Groups C and D were further divided into those terminated 24 h after receiving one dose of Kencur ethanolic extract (addressed as C-24 and D-24 from here onwards) and those terminated after two doses of the extract (C-48 and D-48). In the C-48 and D-48 subgroups, each dose of the extract was separated by 24-h intervals. The grouping scheme and the number of samples in each respective group are shown in Figure 1.

All rats were acclimatised for 7 days by being treated in cages at a stable temperature of 22 ± 2 °C with normal light-dark cycle exposure every 12 h, and were fed the same food and drink. Group A was terminated immediately after the acclimatisation process, while Groups B-24 and B-48 were terminated 24 h and 48 h after trauma onset, respectively. Groups C-24 and D-24 received therapy immediately after trauma induction and were terminated 24 h later. Groups C-48 and D-48 received two doses of therapy: immediately after trauma and after the first 24 h and were terminated 24 h after the second dose (48 h after the onset of trauma).

Subjects were terminated by being decapitated using the cervical dislocation method under general anaesthesia. This method was chosen because it reduces the risk of prolonged hypoxia in the termination process, which can introduce bias to the process of caspase-3 activity.

### Traumatic Brain Injury Induction

After induction of anaesthesia, the rat was positioned under a weight drop device. A scalp incision was made longitudinally along the midline until the skull was exposed. The coronal suture was identified to locate the impact area that was situated 2 mm behind the suture. A stainless-steel helmet of 10 mm in diameter and 3 mm in thickness was mounted into the skull using bone glue. The falling height of the free-falling weight with 450 g brass was set at 100 cm. The impact then hit the helmet, which produced diffuse traumatic brain injury (8–10). The incision was subsequently treated with topical antibiotics to prevent infection problems and was sutured layer by layer.

### Extraction of Kaempferia Galanga L

Kencur (*Kaempferia galanga* L.) was sliced and dried at a temperature of < 70 °C for 24 h and subsequently pulverised into simplicia. The simplicia was then macerated, rotatory-evaporated and mixed with 100 mL of 70% ethanol solvent. Finally, the obtained extract was mixed with 0.5% sodium carboxymethyl-cellulose (CMC) and diluted into a 100 mL solution. Fresh extracts were stored at room temperature using closed tubes away from light exposure. Additional cover was used to further ensure the extracts’ safety from light exposure. The extract was produced as a 60 mg/cc solution.

The extracts were administered orally using a 10 cc syringe connected to a rat feeding tube. For the doses of 600 mg/kgBW/day and 1,200 mg/kgBW/day, we gave 3 cc of extract (equivalent to 180 mg) and 6 cc (equivalent to 360 mg), respectively. Because each rat’s maximum stomach volume is 5 cc, any volume exceeding this capacity was divided into two doses separated by a 30-min interval. The extraction was performed at the pharmacy laboratory of Universitas Airlangga.

### Tissue Sampling

Tissue from brain slice samples (sagittal cut of the cerebrum) was prepared with haematoxylin and eosin (H&E) stain. Immunohistochemistry (IHC) of caspase-3 (E-8: sc-7272 Santacruz Biotechnology Inc.) was performed with a 1:200 dilution. Caspase-3 expression was evaluated using the immunoreactive score (IRS). Based on the IRS, positive cell scores were obtained by multiplying the percentage of positive cell score with the staining intensity of the cell. The percentage of positive cell score was categorised into 0 = no positive cells, 1 = positive cells less than 30%, 2 = positive cells 30%–60% and 3 = positive cells more than 60%. The staining intensity of the cell was categorised into 0 = no colour reaction, 1 = light intensity, 2 = medium intensity and 3 = strong intensity. The pathologist who examined the samples was unaware of the group allocation to reduce the risk of bias in the subjective assessment by the pathologist.

### Statistical Analysis

The data were processed descriptively and analytically. The data in this study were numerical data from more than two unpaired groups. The data was not found to be normally distributed in any of the groups based on the Shapiro-Wilk test (*P* < 0.05). Hence, the comparative analysis for each group was conducted using the non-parametric Kruskal-Wallis test, followed by the Mann-Whitney U test (11). The research data were tested for significance using SPSS version 26.0, with a significance level of 5% (*P* = 0.05).

## Results

**Figure 2:**
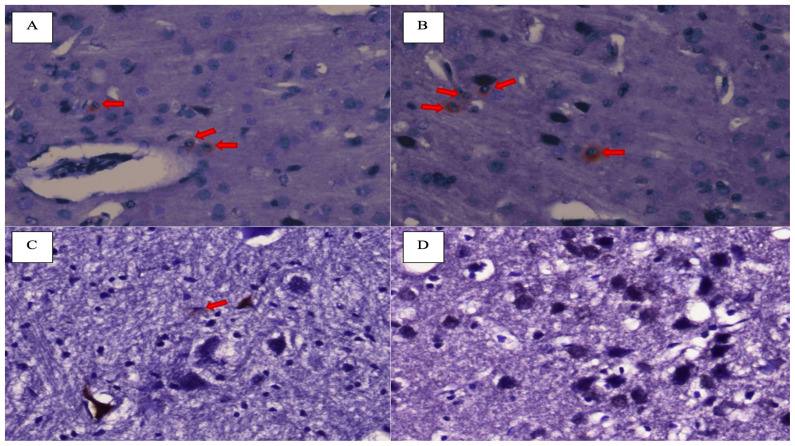
Neuronal apoptosis of the subjects, as indicated by the caspase-3 precipitate around the cell (red arrow). A and B (600× magnification) are Injury groups at 24 h and 48 h after injury without treatment of *Kaempferia galanga* L. extract, respectively. C and D (400× magnification) are Intervention groups with doses of 600 mg/kgBW/day and 1200 mg/kgBW/day at 48 h after injury, respectively. There was no brownish precipitate in D

**Figure 3:**
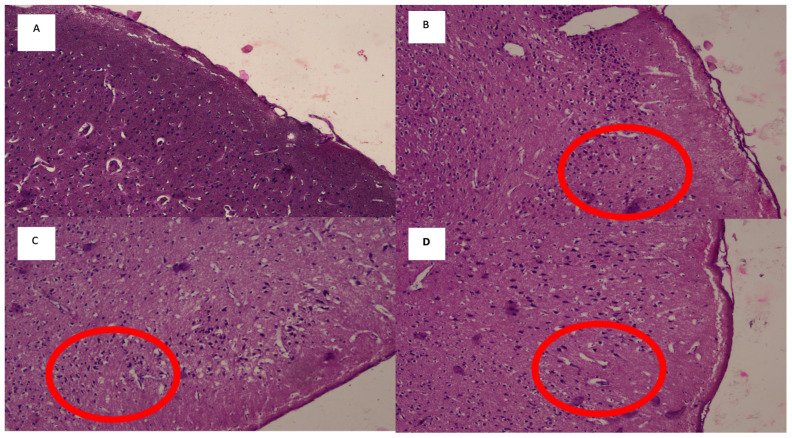
Features of cerebral oedema (100× magnification) with loosening of the interstitial matrix in the subcortical frontal area of the H&E-stained sample: (A) Normal matrix of frontal subcortex area. (B, C and D) Loosening of the interstitial matrix (red circle)

Brownish deposits surrounding the cells in the sample suggested the presence of caspase-3 activity (Figure 2). On evaluation of H&E staining, loosening of the interstitial matrix in the subcortical of the frontal area was found in many samples, which represents cerebral oedema (Figure 3). Therefore, we carried out a comparison test for each group on the expression of caspase-3 and the appearance of cerebral oedema on H&E staining.

A non-parametric analysis of all groups using the Kruskal-Wallis test yielded a P-value of 0.007 for caspase-3 expression and 0.013 for cerebral oedema, indicating the presence of a statistically significant difference between the comparison groups. Analysis using the Mann-Whitney U test was then performed (Table 1).

Statistical analysis (Table 1) showed a significant difference only between the Injury group (Group B-48) and the Intervention group (Group D-48) for caspase-3 expression (*P* = 0.016). There was also a significant difference between the two doses of intervention (C-24 and D-48; P = 0.016 for both caspase-3 and cerebral oedema).

We found significant differences between Group A and Group B-24 in terms of cerebral oedema (*P* = 0.016) but not in caspase-3 expression. There was also a significant difference between Groups A and B-48 with a *P*-value of 0.016 for caspase-3 and 0.032 for cerebral oedema.

In Group D-48, which received two doses of 1,200 mg/kgBW/day, *Kaempferia galanga* L. ethanol extract showed a decrease in the expression of capase-3 in comparison to the Injured group that did not receive therapy (*P* = 0.016). Significant differences were also found between those treated at 1,200 mg/kgBW/day for 48 h (D-48) and those treated at 600 mg/kgBW/day for 24 h (C-24) (*P* = 0.016).

## Discussion

The aim of this study was to analyse the effect of *Kaempferia galanga* L. ethanolic extract treatment on the expression of caspase-3 in traumatic brain injury rat models. The ethanolic extract of *Kaempferia galanga* L. contains active antioxidant and anti-inflammatory ingredients in the form of kaempferol (tetrahydroxyflavone), ethyl p-methoxycinnamate, saponins and ascorbic acid (5–7), which can inhibit free radicals and inflammatory processes in primary and secondary brain injury through the caspase pathway.

The results of this study showed that caspase-3 expression increased significantly 48 h after the injury, which is consistent with the findings of Harter et al. (12), who found that the expression of caspase-3 in patients with traumatic brain injury increased significantly on the second day (48 h) after the injury, peaked on the 5th day and persisted until the 14th day.

The expression of caspase-3 was found to be decreased in the group that received two doses of *Kaempferia galanga* L. ethanol extract at a dose of 1,200 mg/kgBW/day (Group D-48). The median immunoreactive scores (IRS) value was 1.000 (IQR = 2.000) compared to the Injury group that was not given therapy (median IRS = 2.000; IQR = 1.000; *P* = 0.016). However, it was not significant in the Therapy group at a dose of 600 mg/kgBW/day (Groups C-24 and C-48) or in the single dose of 1,200 mg/kgBW/day (Group D-24). There were significant differences in the expression of caspase-3 and cerebral oedema when treated with *Kaempferia galanga* L. ethanolic extract at higher doses (1,200 mg/kgBW/day) within 48 h compared to those treated with half the dose within 24 h. Based on this statistical analysis, it can be concluded that *Kaempferia galanga* L. ethanolic extract has a significant effect on caspase-3 expression and cerebral oedema at higher doses and after a longer duration of treatment.

In 2002, Kurniawan et al. (13) found that brain derived neurotrophic factor expression was significantly different between rats treated with *Kaempferia galanga* L. ethanolic extract at doses of 600 mg/kgBW/day and 1,200 mg/kgBW/day for 48 h compared to those that were not treated. A previous study by Sagita et al. (14) also showed significant differences in the expression of MDA (malondialdehyde) in rats with trauma treated with *Kaempferia galanga* L. ethanolic extract compared to those that were not treated with this extract. Another study by Niantiarno et al. (9) on the expression of aquaporin-4 (AQP4) as a water channel protein and a key regulator of water metabolism in the brain found that the intensity of AQP4 increased in rats with traumatic brain injury, and this expression decreased after treatment with *Kaempferia galanga* L. ethanolic extract at a dose of 1,200 mg/kgBW/day within 24 h and 48 h.

Based on the results of the present study and the findings of previous studies, a conclusion can be drawn that *Kaempferia galanga* L. ethanolic extract might be beneficial in inhibiting caspase-3 expression and alleviating cerebral edema only after a certain time point at a higher dose. Research by Machin et al. (15) showed that using green tea extract containing the antioxidant eppigallocathechin-3-galleate (EGCG) for 7 days could inhibit the process of apoptosis, with significant changes in the expression of caspase-3 and B-cell lymphoma2 (BCL2) in the group receiving therapy at doses of 30 mg/kgBW. However, it was not significant in the lower dose group.

Based on this study, further research could consider the range of evaluation time and *Kaempferia galanga* L. ethanolic extract therapy could be given more than 48 h after trauma. Evaluation of the apoptotic process can also be considered using the terminal deoxynucleotidyl transferase dUTP nick-end labeling (TUNEL) assay and BCL2 methods to provide more representative results.

## Conclusion

The expression of cerebral caspase-3 increased significantly within 48 h after traumatic brain injury. Treatment with Kencur ethanol extract at a dose of 1,200 mg/kgBW/day administered within 48 h can significantly suppress cerebral caspase-3 expression in rats that have experienced traumatic brain injury. Administration of Kencur ethanol extract at a dose of 1,200 mg/kgBW/day for 48 hours is better than a dose of 600 mg/kgBW/day given for 24 h and 48 h or a dose of 1,200 mg/kgBW/day only given for 24 h in suppressing cerebral caspase-3 expression in rats that experienced traumatic brain injury.

## Figures and Tables

**Table 1:** Mann-Whitney U comparison test for caspase-3

Group	Median	IQR	Group	Median	IQR	*P-value*

	Control group (A) compared to Control group with injury (B)					
A	0.000	1.000	B-24	2.000	3.500	0.095
			B-48	2.000	1.000	0.016*

**Control group with injury (B) compared to Therapy groups (C and D)**						

B-24	2.000	3.500	C-24	2.000	0.500	0.841
			D-24	2.000	0.500	0.841
			C-48	2.000	0.500	0.841
			D-48	1.000	2.000	0.151

**Control group with injury (B) compared to Therapy groups (C and D)**						

B-48	2.000	1.000	C-24	2.000	0.500	0.690
			D-24	2.000	0.500	0.690
			C-48	2.000	0.500	0.690
			D-48	1.000	2.000	0.016*

**Comparison between the Therapy groups (C and D)**						

C-24	2.000	0.500	D-24	2.000	0.500	1.000
			C-48	2.000	0.500	1.000
			D-48	1.000	2.000	0.016*

Notes:

* signifies statistical significance;

Median = median value of caspase-3; IRS = immunoreactive score; IQR = interquartile range
